# Role of nuclear factor of activated T cells 2 (NFATc2) in allergic asthma

**DOI:** 10.1002/iid3.360

**Published:** 2020-10-20

**Authors:** Marielena Jakobi, Alexander Kiefer, Hooman Mirzakhani, Manfred Rauh, Theodor Zimmermann, Paraskevi Xepapadaki, Barbara Stanic, Mubeccel Akdis, Nikolaos G. Papadopoulos, Benjamin A. Raby, Scott T. Weiss, Susetta Finotto

**Affiliations:** ^1^ Department of Molecular Pneumology, Universitätsklinikum Erlangen Friedrich‐Alexander‐Universität Erlangen‐Nürnberg Erlangen Germany; ^2^ Department of Allergy and Pneumology, Children's Hospital, Universitätsklinikum Erlangen Friedrich‐Alexander‐Universität (FAU) Erlangen‐Nürnberg Erlangen Germany; ^3^ Channing Division of Network Medicine, Department of Medicine, Brigham and Women's Hospital Harvard Medical School Boston Massachusetts USA; ^4^ Department of Allergy, 2nd Pediatric Clinic National and Kapodistrian University of Athens Athens Greece; ^5^ Swiss Institute of Allergy and Asthma Research (SIAF) University of Zurich Davos Wolfgang Switzerland; ^6^ Centre for Respiratory Medicine and Allergy University of Manchester UK

**Keywords:** allergy processes, animals, eosinophils cells, human, T cells

## Abstract

**Background:**

We recently described increased *NFATc1, IRF4*, and NIP45 messenger RNA (mRNA) expression in peripheral blood mononuclear cells (PBMCs) of asthmatic children and adults with multiple allergies.

**Objective:**

NFATc2 has been described to associate with IRF4 to induce interleukin‐4, and to be inhibited by T‐bet. Here, we analyzed the role of NFATc2 in asthmatic children and adults.

**Methods:**

PBMCs were isolated from the blood of control of asthmatics subjects. Some PBMCs were analyzed untreated and some cultured with and without phytohemagglutinin. Then, RNA was extracted from the cells and cytokines were measured in the supernatants via enzyme‐linked immunosorbent assay or multiplex analysis. RNA was then reverse‐transcribed and NFATc1, NFATC2, IRF4, and T‐bet mRNA were analyzed by real‐time polymerase chain reaction. In addition, in peripheral blood cells, NFATc2 expression was analyzed, in a population of asthmatic children and adults from the Asthma BRIDGE study.

**Results:**

In addition to NFATc1 and NIP45, also NFATc2 was found upregulated in PBMCs and peripheral blood cells from asthmatic children and adults with allergic asthma. Moreover, NFATc1 directly correlated with lymphocytes number whereas NFATc2 correlated with peripheral eosinophilia in asthma.

**Conclusions:**

In addition to NFATc1 and NIP45, NFATc2 was found upregulated in asthma. Moreover, NFATc1 mRNA correlated with lymphocytes both in control and asthma, and NFATC1 and NFATc2 mRNA showed a direct correlation with eosinophils in controls but not in asthma, indicating that NFATc1 is associated with lymphocytes and not eosinophils in asthma.

**Clinical Significance:**

Targeting NFATc2 in T lymphocytes might ameliorate the allergic phenotype in asthmatic subjects.

## Graphical abstract

In addition to NFATc1 and NIP45, NFATc2 was found upregulated in asthma. Moreover, NFATc1 messenger RNA (mRNA) correlated with lymphocytes both in control and asthma. NFATc2 mRNA showed a direct correlation with eosinophils both in controls and asthma, indicating that NFATc1 is associated with lymphocytes and not eosinophils in asthma.

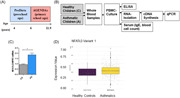

## 1 INTRODUCTION

In allergic asthma, allergen induces interleukin‐4 (IL‐4) increased secretion by lung cells of the innate immune system, like innate lymphoid cells type 2, resulting in chronic differentiation of Th2 cells, eosinophil recruitment into the lung, induction of both memory B cells and long‐lived plasma cells producing high‐affinity IgE antibodies that bind to the high‐affinity receptor expressed on the surface of mast cells, basophils, and eosinophils.^1^ These cells then get pathological activated and release proinflammatory mediators that cause the classic characteristics of asthma.

Nuclear factor of activated T cells c1 (NFATc1) belongs to a family of transcription factors that are known to be activated by calcium influx. The different family members play different roles in T cell development and activation and thus NFAT inhibitors have been used during organ transplantation to avoid transplant rejection as well as to control autoimmune diseases. It has been suggested that NFATc2 together with NFATc1 is crucial for T cell differentiation. We have previously demonstrated *NFATc1* messenger RNA (mRNA) induction in peripheral blood mononuclear cells (PBMCs) in pediatric asthma and that the absence of NFATc1 in T cells results in the downregulation of IL‐4 and other Th2 cytokines and IgE in asthma models.^2^, ^3^, ^4^ We also recently reported that the NFAT‐interacting protein (NIP)‐45, which induced IL‐4 by potentiating NFATc2, is induced in preschool children with asthma and NIP45 deficient mice have less airway eosinophilia.^2^

Here, we sought to investigate the role of NFATc1 and NFATc2 in different cohorts of asthmatic and control children and adults to better understand the distinct role of these two transcription factors and their association with different forms of asthma.

## 2 METHODS

### 2.1 Human study PreDicta

The European Study PreDicta (postinfectious immune reprogramming and its association with persistence and chronicity of respiratory allergic diseases) has been performed in parallel in different pediatric centers in Europe (Predicta study No. 260895 of EU FP7). In this study, we further examined the PreDicta longitudinal Cohort WP‐1 composed of 21 healthy and 24 asthmatic preschool children at the age of 4–6 years in collaboration with the Department of Allergy and Pneumology at the Children Hospital at the FAU University Hospital in Erlangen.

The study in Erlangen was approved by the local ethics committee of the Friedrich‐Alexander University Erlangen‐Nürnberg, Germany (Re‐No 4435) and it is registered in the German Clinical Trials Register (www.germanctr.de: DRKS00004914).

The recruitment of the subjects, inclusion and exclusion criteria, as well as the timescale for clinical visits and data collection, were recently described along with their clinical characteristics.

### 2.2 Additional cohorts

#### 2.2.1 Asthma BRIDGE and CAMP study cohorts

For replication of findings in the PreDicta cohort, that is, higher expression of NFATC2 in the PBMC of asthmatic children, we used microarray gene expression data from the Asthma Biorepository for Integrative Genomic Exploration (Asthma BRIDGE, *N* = 1448) and Childhood Asthma Management Program (CAMP, *N* = 620; Table S1).^5^, ^6^ In both cohorts, asthma was defined based on having a physician diagnosis of the disease with evidence of reversible airflow obstruction with a bronchodilator. Total of 981 subjects (865 with asthma) had whole blood (WB) expression data in both cohorts who were used for these studies (range of age, 5–32 years old).

##### 2.2.1.1 Expression analysis of NFATC2 in WB

All subjects from Asthma BRIDGE and CAMP WB had gene expression data measured using Illumina HumanHT‐12 v4 Expression BeadChip (Illumina, Inc.) that passed stringent and commonly used quality control metrics.^6^ We used the R Bioconductor limma package (version 3.42) to perform differential expression analysis. A linear model was fitted along with the implementation of empirical Bayes statistics on log_2‐_transformed and quantile‐normalized gene expression (*n* = 47,009 probes) to assess whether the expression level of the candidate gene NFATC2 was significantly altered among asthmatics compared with controls (*p* < .05). The differential gene expression model was adjusted for three important confounders (age, sex, and self‐reported race) as well as the batch effect.

### 2.3 Gender, age, and environmental factors that modify immune‐response and the development of allergic asthma during the school age in childhood (AGENDAS)

This is a new study, acronym AGENDAs (which has been initiated in Erlangen and the recruitment continues to date with the same team that performed the PreDicta study and using the same clinical and experimental protocols. In this longitudinal study, seven healthy and six asthmatic primary school‐aged children at the age between 6 and 11.9 years were analyzed to date in collaboration with the Department of Allergy and Pneumology at the Children Hospital at the FAU University Hospital in Erlangen.

The study in Erlangen was approved by the local ethics committee of the Friedrich‐Alexander University Erlangen‐Nürnberg, Germany (Re‐No: 212_12 B) and it is registered in the German Clinical Trials Register (www.germanctr.de: DRKS00004914).^1^, ^3^, ^6^, ^7^

The recruitment of the subjects, inclusion and exclusion criteria as well as the timescale for clinical visits, and data collection were recently described for the cohort Predicta.^1^, ^3^, ^6^, ^7^

### 2.4 FEV1 and peak expiratory flow

Forced expiratory volume at 1 s (FEV1), forced vital capactity (FVC), and peak expiratory flow were measured at the baseline visit (B0) by using spirometry. After a period of normal breathing, the participant should inhale maximal, directly followed by maximal and fast exhalation. The volume exhaled in 1 s is FEV1. The total exhaled volume is FVC. The ratio FEV1/FVC is stated as Tiffenau Index. A child during spirometry will be asked to inhale as deep as possible before exhaling as strong and fast as possible for 6 s in total. This was repeated at least three times depending on the child's capacity to follow the instructions properly. Tiffenau Index measures the volume of breath in the first second of expiration compared with its vital capacity. This is a widely used measure for the severity of obstruction and comparable amongst individuals.

### 2.5 Radioimmunoassay test

This is a blood test using radioimmunoassay to detect allergen‐specific IgE antibodies directed against allergens, to determine the substance a subject is allergic to.

Specific IgE levels were measured by means of the ImmunoCAP system (Thermo Fisher‐Phadia) as instructed by the manufacturer. According to the manufacturer instructions, the specific IgE level more than 0.35 kU/L was considered as positive (stated as RK1 and above).

### 2.6 Differential blood cell count

Complete blood cell count analyses were performed on Sysmex analyzer (XN‐350, XN‐1000, SYSMEX Deutschland) in accordance with standard operating procedures.

### 2.7 Isolation of human PBMCs, in vitro cell culture from the children cohorts

At the time of recruitment (Baseline Visit), PBMCs were isolated from heparinized blood with Ficoll using density centrifugation. After isolation, the PBMC number was adjusted to a concentration of 10^6^ viable cells/ml in the complete culture medium. For cell culture, Roswell Park Memorial Institute 1640 medium supplemented with 25 mmol/L HEPES (GIBCO, Invitrogen) was used. Furthermore, 100 IU/ml penicillin, 100 µg/ml streptomycin, 50 µmol/L β‐mercaptoethanol, and 1% l‐glutamine (200 mmol/L), 1% minimal essential medium vitamin, 1% nonessential amino acids, 1% sodium pyruvate, and 10% fetal bovine serum were added (complete culture medium); these reagents were purchased from Sigma‐Aldrich. The PBMCs were cultured in a complete culture medium for 24 h at 37°C and 5% CO_2_, whereby parts of them were challenged in vitro with or without phytohemagglutinin (PHA‐M) at the final concentration of 10 mg/ml. At the end of the cell culture, cell supernatants were collected separately and stored frozen for further cytokine analysis. The cell pellet was incubated in PeqGOLD and later on RNA extracted as previously described. For gene expression analysis, we extracted mRNA and performed quantitative real‐time polymerase chain reaction (RT‐PCR).^7^

### 2.8 Enzyme‐linked immunosorbent assay

Human transforming growth factor‐β1 (TGF‐β1) was detected in the cell‐culture supernatants by using the TGF‐β ELISA kit from PeproTech according to the manufacturer's protocol.

### 2.9 Luminex

It is a Luminex x‐MAP technology (x = analyte, MAP = multianalyte profiling)‐based 22‐plex human cyto/Th17 Magnetic Bead Panel (from EMD, Millipore). The samples were thawed, and the procedure followed the manufacturer's instructions for the quantitative assessment of the cell culture supernatant‐type of samples (incubation during an overnight period [12 h]). The analysis was done using Luminex 200 machine. The concentration of analytes in samples was expressed as pg/ml.

### 2.10 RNA isolation and quantitative RT‐PCR

Total RNA was extracted from PBMCs by using PeqGold RNA Pure according to the manufacturer's protocol (PeqLab). Afterward, 1 µg RNA was reverse‐transcribed using the first‐strand complementary DNA (cDNA) synthesis kit for RT‐PCR (MBI Fermentas). The resulting cDNA was amplified by quantitative RT‐PCR using SsoFast EvaGreen Supermix (Bio‐Rad Laboratories). The qPCR was performed with a cycle of 2 min 98°C, 50 cycles at 5 s 95°C, 10 s 60°C, followed by 5 s 65°C, and 5 s 95°C in a CFX96 Touch Real‐Time PCR Detection System (Bio‐Rad Laboratories). The primers and sequences used are reported below

**Table:** 

NFATc1 fw	5′‐GCA TCA CAG GGA AGA CCG TGT C‐3′
NFATc1 rev	5′‐GAA GTT GAA TGT CGG ATG TTC TGA G‐3′
NFATc2 fw	5′‐ACA ACA TGA GGG CAA CCA TCG‐3′
NFATc2 rev	5′‐GTC CAT CTG TGG TCT TCT CAG‐3′
HPRT fw	5′‐TGA CAC TGG CAA AAC AAT GCA‐3′
HPRT rev	5′GGT CCT TTT CAC CAB CAA GCT‐3′
Tbet fw	5′‐CCG TGA CTG CCT ACC AGA ATG‐3′
Tbet rev	5′AAC AGG ATA CTG GTT GGG TAG GA‐3′
hIRF4 fw	5′‐AGA CTG TGC CAG AGC AGG AT‐3′
hIRF4 fw	5′‐GGG TCT GGA AAC TCC TCT CC‐3′
FOXP3 fw	5′‐AAC AGC ACA TTC CCA GAG TTC CT3′
FOXP3 rev	5′‐CAT TGA GTG TCC GCT GCT TCT ‐3′
PD1 fw	5′‐CAG TTC CAA ACC CTG GTG GT‐3′
PD1 rev	5′‐GGC TCC TAT TGT CCC TCG TG‐3′

### 2.11 Statistical analysis

Statistically significant (*p* < .05) differences were evaluated by a Student's unpaired *t*‐test for independent events by using GraphPad Prism version 8.1.2 for Windows, (GraphPad Software). The *p*‐value for the *t*‐test linear regression in the correlations is indicated as two‐tailed by using the same computer program. Data are shown as mean values ± *SEM*.

## 3 RESULTS

**Figure 1:**
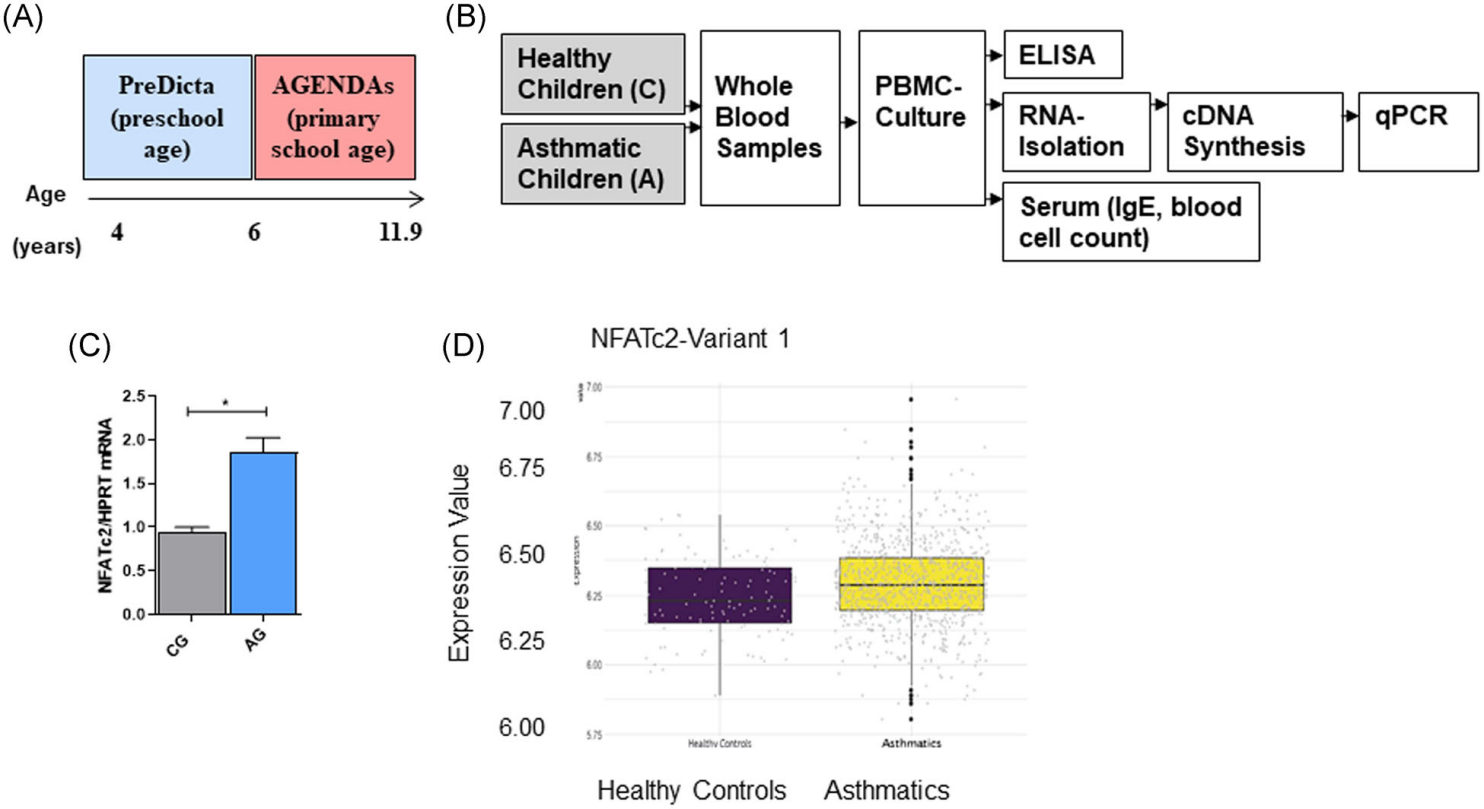
*NFATc2* is induced in PBMCs of preschool children as well as in peripheral blood cells from asthmatic children and adults with allergic asthma. (A) Age range in the PreDicta cohort compared with AGENDAs. In the PreDicta cohort we examined children at preschool age. (B) Experimental design of the PreDicta and AGENDAs studies (C) *NFATc2* mRNA measured by qPCR in untreated PBMCs isolated from the PreDicta cohorts: control group (CG); *n* = 2 and asthma group (AG); *n* = 10. *p* = .0125 unpaired *t*‐test. (D) Increased mRNA expression of *NFATc2* in peripheral blood of asthmatic children and adults compared with healthy controls in Asthma Bridge and CAMP cohorts. We used microarray gene expression data from the Asthma Biorepository for Integrative Genomic Exploration (Asthma BRIDGE, *N* = 1448) and Childhood Asthma Management Program (CAMP, *N* = 620). The shorter isoform A (variant 1) of NFATC2 was significantly overexpressed in peripheral blood from asthmatics (*n* = 865) compared with nonasthmatic, healthy controls (*n* = 865 vs. *n* = 116, respectively; fold‐change = 1.043, *p* = .02). Statistical Significance as indicated: *p ≤ .05; **p ≤ .01; ***p ≤ .001. CAMP, Childhood Asthma Management Program; cDNA, complementary DNA; ELISA, enzyme‐linked immunosorbent assay; mRNA, messenger RNA; NFATc2, nuclear factor of activated T cells c2; PBMCs, peripheral blood mononuclear cells; qPCR, quantitative polymerase chain reaction

In this study, we thus started to investigate the regulation of NFATc2 in the PBMCs in the PreDicta cohorts of preschool children with and without asthma. The clinical characteristics of the two cohorts were previously reported.^7^ The experimental design of PreDicta study is summarized again in Figure 1A,B. Here, we found *NFATc2* mRNA upregulated in untreated PBMCs, from asthmatic children as compared with those from control children (Figure 1C).

Moreover, *NFATc2* mRNA showed altered expression levels in peripheral blood from 981 children and adults from the Asthma BRIDGE and CAMP cohorts (Table S1). The shorter isoform A (variant 1) of *NFATc2* was significantly overexpressed in peripheral blood from asthmatics (*n* = 865) compared with nonasthmatic, healthy controls (*n* = 865 vs. *n* = 116, respectively; foldchange = 1.043, *p* = .02; Figure 1D).^6^, ^8^

We next reasoned, based on our PreDicta data (Figure S1A,B), that NFATc2 might associate with both Th1 and Th2 responses dependent on other transcription factors regulating Th1 and Th2 genes present in the microenvironment. We also looked at TGF‐β1 in PBMCs cultured with and without PHA, to have an idea of immunosuppression in preschool asthma. Here, we found a trend towards the downregulation of activated TGF‐β1 in the supernatants of PBMCs cultured with PHA, isolated from asthmatics preschool children (Figure S1C).

For this reason, we next looked at correlations between NFATc1/c2 and IRF4, a transcription factor present on the promoter of many Th2 and Th9 cytokines and known to interact with NFATc2 on the IL‐4 promoter.^9^ Here we found that *NFATc2* mRNA correlated with *IRF4* mRNA by a trend in PBMCs from control but not in asthmatic children (Figure S1D,E). Moreover, NFATc1 mRNA inversely correlated with NFATc2 mRNA in asthmatic children.

**Figure 2:**
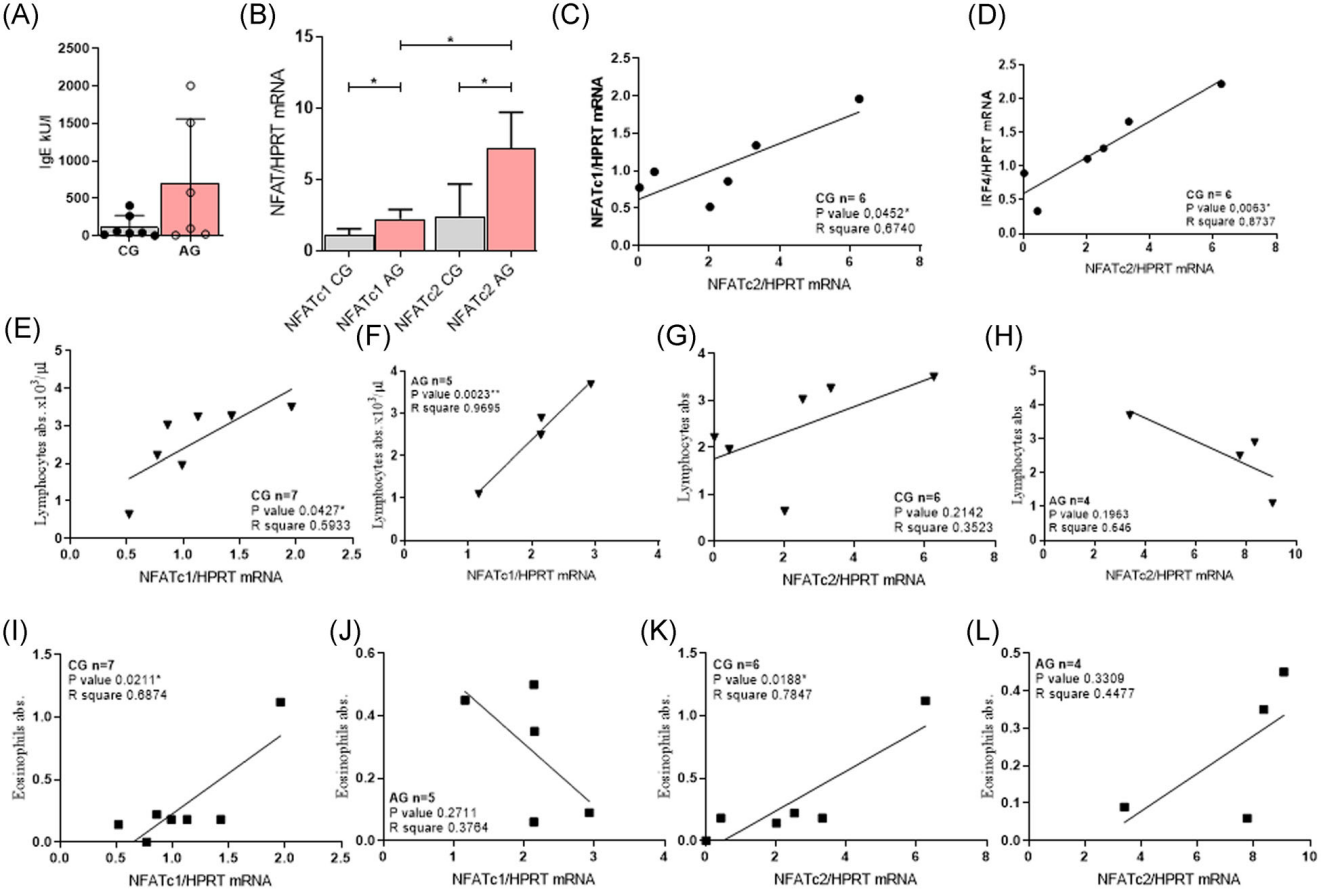
*NFATc1* and *NFATc2* mRNA are upregulated also in asthmatic primary school children and NFATc1 is positively correlated with lymphocytes in peripheral venous blood of asthmatic children. (A) IgE concentration was measured in the peripheral venous blood of the AGENDAs cohorts: CG *n* = 7 AG *n* = 6. (B) *NFATc1* and *NFATc2* mRNA were measured in untreated PBMC culture in the cohort of primary school children AGENDAs. NFATc1: CG *n* = 7; NFATc2: CG *n* = 6. (C) and (D) NFATc2 mRNA correlated with IRF4 mRNA in CG *n* = 6. (E) NFATc1 correlated with lymphocytes from the peripheral venous blood of the CG *n* = 7 and (F) AG *n* = 5. (G) NFATc2 positively correlated with lymphocytes from the peripheral venous blood of the CG *n* = 6 but not AG *n* = 4. (H) and (I) *NFATc1 mRNA* positively correlated with eosinophils from the peripheral venous blood of the CG *n* = 7 but not from and AG *n* = 5 . (J) and (K) *NFATc2 mRNA* correlated with eosinophils of the peripheral venous blood of the CG *n* = 6 and AG *n* = 4  (L). Significance as indicated: *p ≤ 0.05; **p ≤ .01; ***p ≤ .001. mRNA, messenger RNA; NFAT, nuclear factor of activated T cells

We next analyzed NFATc1 and NFATc2 mRNA in the PBMCs in the newly recruited cohorts of children one without asthma (control children, *n* = 7) and the second one with asthma (asthma children, *n* = 6) at primary school‐age (AGENDAs cohort). The clinical characteristics of the allergic trait of these children are reported in Figure 2A and Table S2–S4. Despite the small group size, we still found a significant induction of both NFATc1 mRNA but especially NFATc2 mRNA in the PBMCs of asthmatic children as compared to control children (Figure 2B). Moreover, in PBMCs from control children, NFATc1 mRNA correlated with NFATc2 mRNA in control but not asthmatic children, indicating the presence of alternatively activated pathways in asthmatic children (Figures 2C and S2A). Here, in fact, we found a direct correlation between NFATc2 and IRF4 mRNA in control children (Figure 2D) but not in asthmatic children (Figure S2B). We also looked at TGF‐β1 in PBMCs of these cohorts of children. Indeed, we confirmed the PreDicta data on a not significant trend towards downregulation of activated TGF‐β1 in the supernatants from PBMCs isolated from asthmatics primary school children (Figure S2C).

We next looked at other markers of immunosuppression known to be regulated by TGF‐β and NFAT, like PD1 and FOXp3. Here we could not find specific correlations (Figure S2D–K). Consistently, we did not observe any significant deviation in terms of differential cell numbers between controls and asthmatic children (Figure S3 and Table S5). We next correlated NFATc1 and NFATc2 with peripheral blood lymphocytes and found that *NFATc1* mRNA directly correlated with lymphocytes in controls and asthmatic children (Figure 2E,F, respectively). By contrast, *NFATc2* mRNA showed an inverse correlation with lymphocytes only in asthmatic children, not controls (Figure 2G,H). In addition, no specific trend was observed for neutrophils (Figure S3A–D), basophils (Figure S3E–H), and monocytes (Figure S3I–L), and *NFATc1* and *NFATc2* mRNA.

We next asked if NFATc2 would correlate with eosinophils in asthma as NFATc2 was associated with a Th2 trait. Indeed, we found a direct correlation between *NFATc1* and *NFATc2* mRNA in PBMCs and peripheral blood eosinophils but only in control children (Figure 2I–L). In conclusion, NFATc1 correlated with peripheral lymphocytes and eosinophils and NFATc2 correlated only with peripheral eosinophils in control children. Similarly, in asthma, we found only a direct correlation of NFATc1 with lymphocytes in control children and an opposite correlation between NFATc1 and peripheral eosinophils in asthma. By contrast, NFATc2 showed a direct correlation with eosinophils in control children.

## 4 DISCUSSION

Several transcription factors controlling T cell fate are relevant in allergy and asthma because they regulate downstream cytokines that control peripheral blood cell traffic and differentiation.^10^ The NFATc1 family members are very important in T cell survival as they regulate IL‐2 production.^11^ However, this regulation can be orchestrated by different transcription factors like activator protein‐1 (AP‐1). NFAT interacts with AP‐1 to induce the effector immune response and in the absence of AP‐1 it induces T cell anergy/exhaustion.^12^ This recent study used small molecules that selectively disrupted the interaction of NFAT and AP‐1 without inhibiting their binding to the target promoter, or other small molecules interacted with the transcription factors DNA binding to the IL‐2 promoter indicating that targeting transcription factor could represent new avenues to treat human diseases like allergic asthma without having the generalized immunosuppressive effects of cyclosporin A. In this study, we extended the findings of the upregulation of NFATc1 to different cohorts of children at primary school age. Moreover, we extended our analysis to NFATc2 which was also found upregulated in different cohorts of children and adults with asthma. In addition, we tried to shed light on the relationship between NFATc1 and NFATc2 and IRF4, another transcription factor upregulating cytokines like IL‐4 and IL‐9 involved in allergic asthma. Here, we found in control children a good correlation between NFATc1 and NFATc2 and NFATc2 and IRF4 mRNA from PBMCs. Interestingly, NFATc1 and NFATc2 directly correlated with peripheral eosinophils in these control children. Moreover, NFATc1 and not NFATc2 directly correlated with peripheral lymphocytes in these control children indicating that NFATc2 associated with eosinophils and thus with an allergic trait. Moreover, NFAT is known to induce tolerance by interacting with the transcription factor Foxp3 induced by TGF‐beta.^13^ Although we could not find any statistically significant correlation between Foxp3 and NFATc1/NFATc2 mRNA in these children, we cannot exclude that this is due to the limited number of the children analyzed. Furthermore, extended studies are needed. Thus by switching transcriptional partners, NFAT converts the acute T cell activation program into the suppressor program of Tregs. Furthermore, NFAT family members are expressed in different cell types like myeloid cells which play a central role in regulating tolerance versus inflammation. In fact, it has been described that the calcineurin‐NFAT‐IL‐2 pathway in myeloid cells is a critical regulator of intestinal homeostasis by influencing the balance of inflammatory and regulatory responses in the mouse intestine.^14^

Furthermore, Fc receptor‐like (FCRL) proteins are novel regulators of the B cell response to antigen. Altering FCRL expression could regulate the B cell response to antigen. Specifically, induced FCRL5 expression required elevation of intracellular Calcium and was partially blocked by cyclosporine A, a calcineurin inhibitor. The importance of the transcription factors NF‐kappa B, NFAT, and CREB‐binding protein was revealed based on mutations of two predicted that NF‐kappa B or an NFAT binding sites in the core promoter abrogated induced gene expression, suggesting direct regulation of the FCRL5 gene by NF‐kappa B and NFAT.^15^, ^16^, ^17^

In summary, the complexity of the transcription factors network cannot be resolved by the downregulation of a single transcription factor. However, the different role of the NFATc family members needs to be taken into consideration in future extensive studies especially in human diseases.

In summary, in this paper, we started to analyze the correlation between NFATc1 and NFATc2 in asthma with different cells present in the peripheral blood of children with and without asthma. Here, we found that *NFATc1* mRNA correlated with lymphocytes both in control and asthma and both *NFATc1* and *NFATc2* mRNA showed a direct correlation with eosinophils in controls but not in asthma, indicating that NFATc1 is associated with lymphocytes and not eosinophils in asthma.

Recently, two types of eosinophils have been described, one inflammatory induced type and supported by IL‐5 and the resident type involved in the tissue homeostasis.^18^ In this manuscript, we found that *NFATc2* mRNA positively correlated with IL‐5 in asthma. Consistently, in the AGENDAs cohort, *NFATc2* mRNA correlated with peripheral blood eosinophils in asthma. It is thus possible that NFATc2 correlates with inflammatory eosinophils whereas NFATc1 correlates with lymphocyte proliferation in asthma. In summary, this study just starts to investigate different roles of NFATc1 and NFATc2 in allergic asthma whereby we found that NFATc2 relates more closely to peripheral blood eosinophils and NFATc1 to lymphocyte proliferation in pediatric asthma. Since allergic asthma is a multifactorial disease, a subtype of the disease might be treated with a tailored therapy targeting different NFAT family members.

## CONFLICT OF INTERESTS

The authors declare no conflict of interest on the matter described in this manuscript.

## AUTHOR CONTRIBUTIONS

Marielena Jakobi measured, correlated, analyzed, and interpreted the data for Figures 1 and 2 and S1–S3, generated Tables S2–S5, and wrote part of the methods and results section, was involved in drafting the manuscript, and edited it. Alexander Kiefer recruited, analyzed, and did the diagnosis of the children from the WP1 Predicta Cohort in Erlangen in PreDicta Study and recruited, analyzed, and made a diagnosis of the AGENDAs cohorts and edited the manuscript. Hooman Mirzakhani analyzed the data in CAMP and ABRIDGE and generated Figure 1D and Table S1 and wrote the relative Methods, Results, and Figure legends, and edited the manuscript. Manfred Rauh generated the data of the differential blood cell count and the RAST data. Mubeccel Akdis and Barbara Stanic provided the Multiplex cytokines data for the Erlangen WP1 Cohort Predicta reported and correlated in Figures S1A and S1B. Nikolaos G. Papadopoulos was the coordinator of the Predicta studies and Paraskevi Xepapadaki helped Nikolaos G. Papadopoulos to the establishment of the different European WP1 cohorts in Predicta. Benjamin A. Raby and Scott T. Weiss were involved in the recruitment and analysis of the CAMP and Asthma BRIDGE studies and mentored Hooman Mirzakhani for Figure 1D and Table S1 analysis and edited the manuscript. Susetta Finotto wrote the manuscript, analyzed, and mentored all the study for the Predicta and AGENDAs part in Erlangen.

## Supporting information

Supporting information.

Supporting information.

Supporting information.

Supporting information.

Supporting information.

## Data Availability

Data available on request due to privacy/ethical restrictions. The data that support the findings of this study are available on request from the corresponding author. The data are not publicly available due to privacy or ethical restrictions

## References

[1] Gould HJ , Sutton BJ . IgE in allergy and asthma today. Nat Rev Immunol. 2008;8:205‐217. 18301424 10.1038/nri2273

[2] Koch S , Reppert S , Finotto S . NFATc1 deletion in T lymphocytes inhibits the allergic trait in a murine model of asthma. Clin Exp Allergy. 2015;45:1356‐1366. 25640055 10.1111/cea.12493

[3] Ranger AM , Hodge MR , Gravallese EM , et al. Delayed lymphoid repopulation with defects in IL‐4‐driven responses produced by inactivation of NF‐ATc. Immunity. 1998;8:125‐134. 9462518 10.1016/s1074-7613(00)80465-3

[4] Yoshida H , Nishina H , Takimoto H , et al. The transcription factor NF‐ATc1 regulates lymphocyte proliferation and Th2 cytokine production. Immunity. 1998;8:115‐124. 9462517 10.1016/s1074-7613(00)80464-1

[5] Yan X , Chu JH , Gomez J , et al. Noninvasive analysis of the sputum transcriptome discriminates clinical phenotypes of asthma. Am J Respir Crit Care Med. 2015;191:1116‐1125. 25763605 10.1164/rccm.201408-1440OC PMC4451618

[6] Raby BA , Van Steen K , Celedón JC , Litonjua AA , Lange C , Weiss ST . Paternal history of asthma and airway responsiveness in children with asthma. Am J Respir Crit Care Med. 2005;172:552‐558. 15937295 10.1164/rccm.200501-010OC PMC2718530

[7] Bergauer A , Sopel N , Kroß B , et al. IFN‐alpha/IFN‐lambda responses to respiratory viruses in paediatric asthma. Eur Respir J. 2017;49:1700006. 27836955 10.1183/13993003.00969-2016

[8] Keene JD , Jacobson S , Kechris K , et al. Biomarkers predictive of exacerbations in the SPIROMICS and COPD gene cohorts. Am J Respir Crit Care Med. 2017;195:473‐481. 27579823 10.1164/rccm.201607-1330OC PMC5378424

[9] Rengarajan J , Mowen KA , McBride KD , Smith ED , Singh H , Glimcher LH . Interferon regulatory factor 4 (IRF4) interacts with NFATc2 to modulate interleukin 4 gene expression. J Exp Med. 2002;195:1003‐1012. 11956291 10.1084/jem.20011128 PMC2193700

[10] Finotto S , Glimcher L . T cell directives for transcriptional regulation in asthma. Springer Semin Immunopathol. 2004;25:281‐294. 15007632 10.1007/s00281-003-0143-1

[11] Zhang L , Nabel GJ . Positive and negative regulation of IL‐2 gene expression: role of multiple regulatory sites. Cytokine. 1994;6:221‐228. 8054477 10.1016/1043-4666(94)90016-7

[12] Mognol GP , González‐Avalos E , Ghosh S , et al. Targeting the NFAT:AP‐1 transcriptional complex on DNA with a small‐molecule inhibitor. Proc Natl Acad Sci U S A. 2019;116:9959‐9968. 31019078 10.1073/pnas.1820604116 PMC6525529

[13] Sundrud MS , Rao A . New twists of T cell fate: control of T cell activation and tolerance by TGF‐beta and NFAT. Curr Opin Immunol. 2007;19:287‐293. 17433870 10.1016/j.coi.2007.04.014

[14] Wu Y , Borde M , Heissmeyer V , et al. FOXP3 controls regulatory T cell function through cooperation with NFAT. Cell. 2006;126:375‐387. 16873067 10.1016/j.cell.2006.05.042

[15] Mencarelli A , Khameneh HJ , Fric J , et al. Calcineurin‐mediated IL‐2 production by CD11c(high)MHCII(+) myeloid cells is crucial for intestinal immune homeostasis. Nat Commun. 2018;9:1102. 29549257 10.1038/s41467-018-03495-3 PMC5856784

[16] Damdinsuren B , Dement‐Brown J , Li HF , Tolnay M . B cell receptor induced Fc receptor‐like 5 expression is mediated by multiple signaling pathways converging on NF‐kappa B and NFAT. Mol Immunol. 2016;73:112‐121. 27065451 10.1016/j.molimm.2016.04.001

[17] de Gorter DJJ , Vos JCM , Pals ST , Spaargaren M . The B cell antigen receptor controls AP‐1 and NFAT activity through Ras‐mediated activation of Ral. J Immunol. 2007;178:1405‐1414. 17237388 10.4049/jimmunol.178.3.1405

[18] Mesnil C , Raulier S , Paulissen G , et al. Lung‐resident eosinophils represent a distinct regulatory eosinophil subset. J Clin Invest. 2016;126:3279‐3295. 27548519 10.1172/JCI85664 PMC5004964

