# Supplementary figures and images for: Role of nuclear factor of activated T cells 2 (NFATc2) in allergic asthma
Source: Immun Inflamm Dis. 2020 Oct 20;8(4):704–12. doi: 10.1002/iid3.360 (PMC7654396; doi:10.1002/iid3.360)

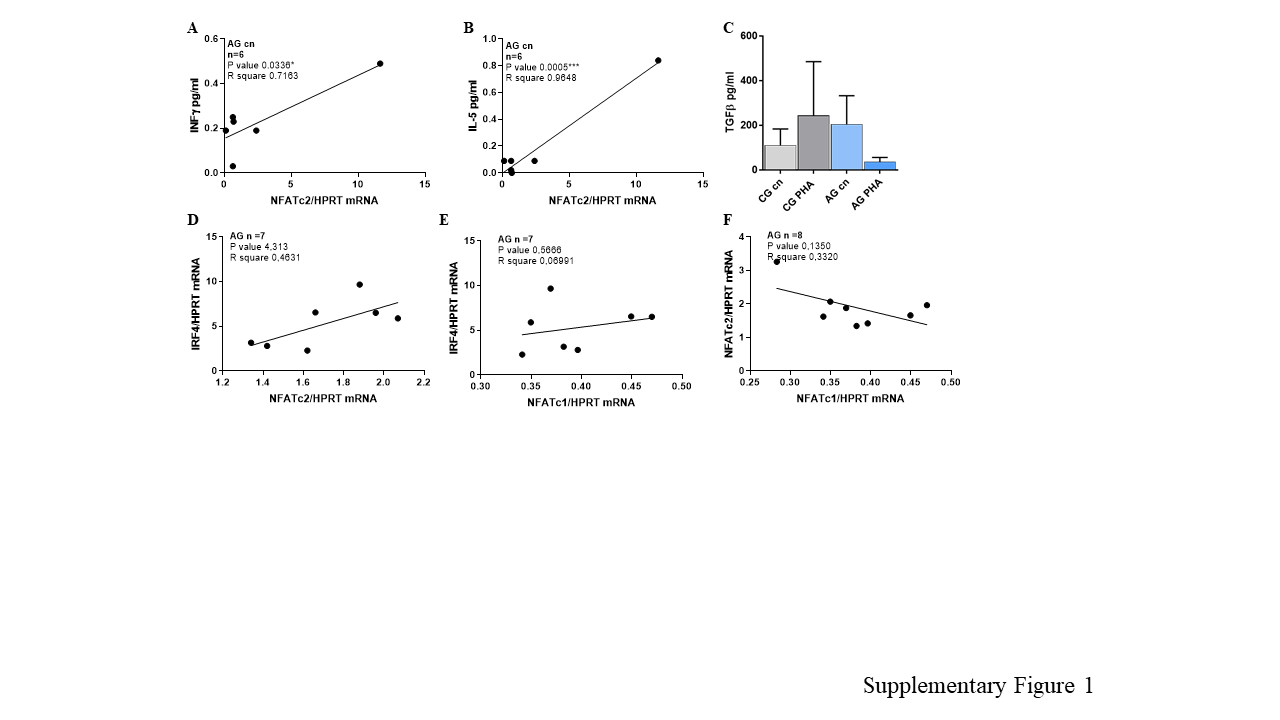

Supplement: Supplementary file 1 — Supporting information. [file IID3-8-704-s001.TIF]

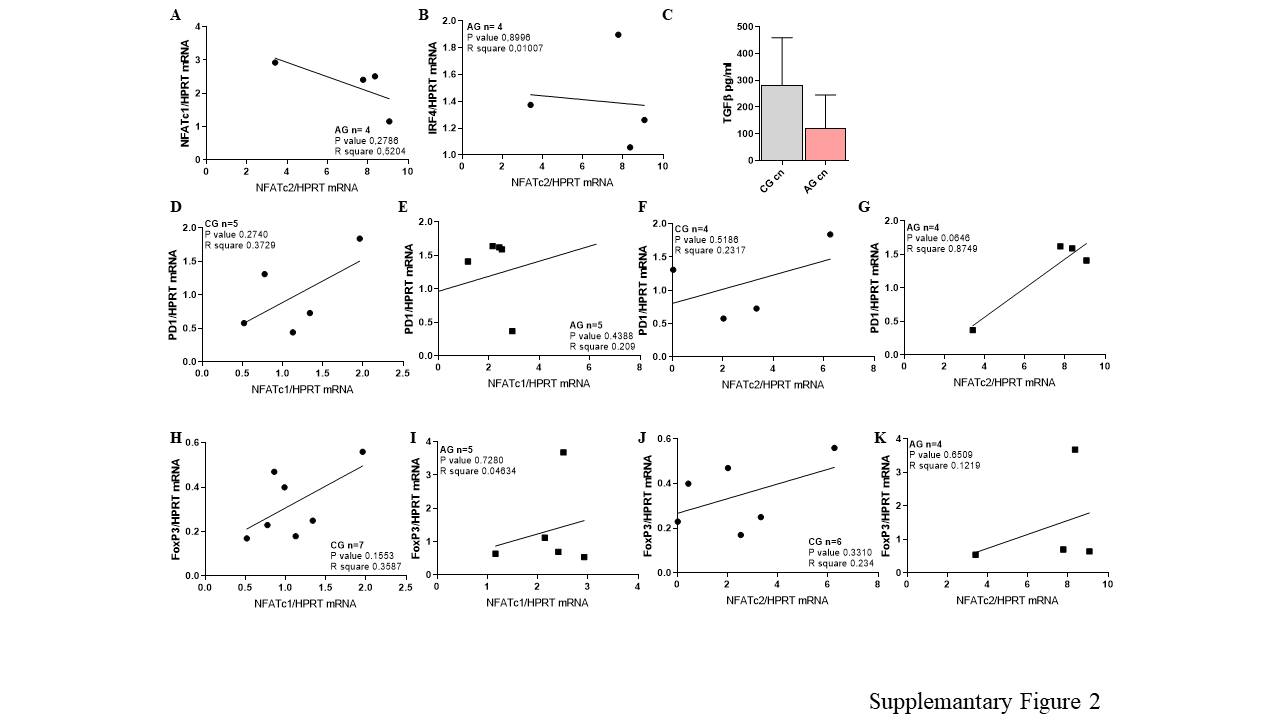

Supplement: Supplementary file 2 — Supporting information. [file IID3-8-704-s002.TIF]

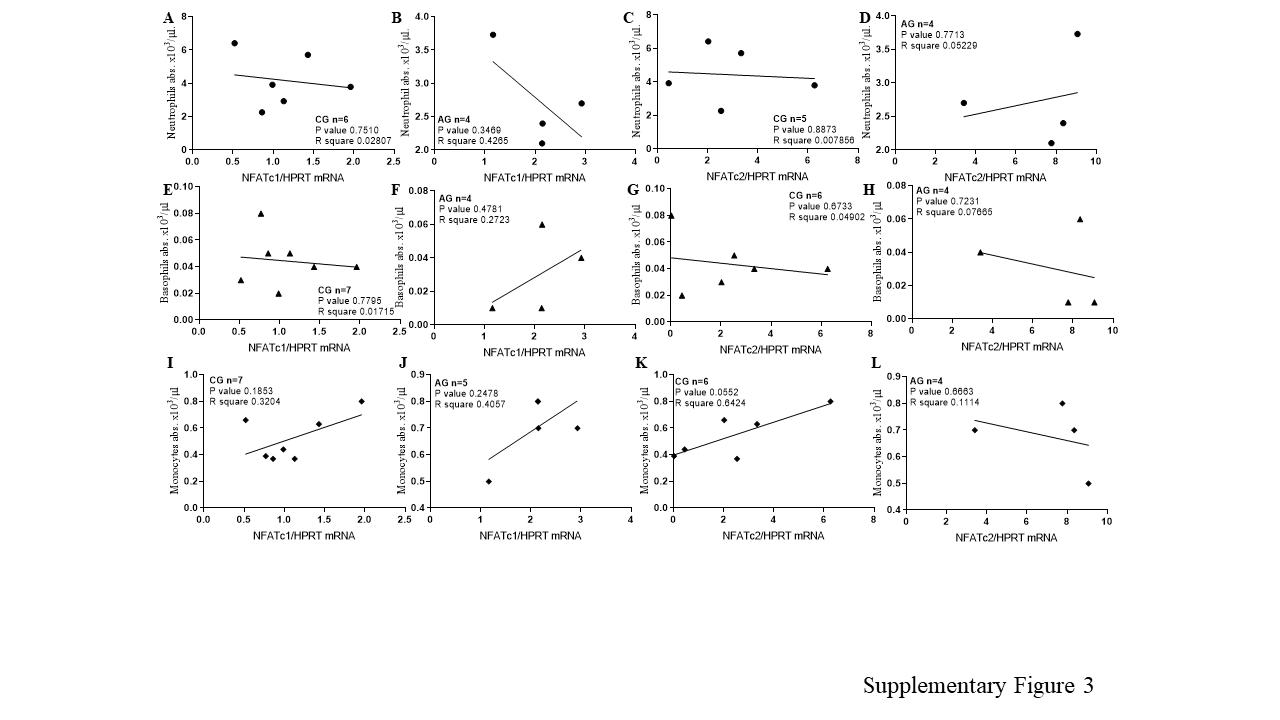

Supplement: Supplementary file 3 — Supporting information. [file IID3-8-704-s003.TIF]
