# Supplementary material for: Role of nuclear factor of activated T cells 2 (NFATc2) in allergic asthma
Source: Immun Inflamm Dis. 2020 Oct 20;8(4):704–12. doi: 10.1002/iid3.360 (PMC7654396; doi:10.1002/iid3.360)
Supplement: Supplementary file 4 — Supporting information. [file IID3-8-704-s004.docx]

**Supplementary Figure Legends**

**Figure S1: Pre-School children PreDicta cohorts in Erlangen. A.** NFATc2 correlated with INFγ in unstimulated PBMC from asthmatic children (AG): p=0.0336, n=6. **B.** NFATc2 correlated with IL-5 in unstimulated PBMCs from asthmatic children:AG p=0.0005 n=6. **C.**TGFβ1 was measured by ELISA in the supernatants of PBMC culture cn (unstimulated PBMC: CG n=16 and AG n=22) and PHA (Phytohemagglutinin stimulated PBMCs: control group -CG n=19 and AG n=20). *NFATc1* and *NFATc2* and *IRF4* mRNA were measured in PBMC in the cohort of asthmatic primary school children AGENDAs . **D.** NFATc2 correlated with IRF4 in PHA stimulated PBMCs in AG in the PreDicta study n=7. **E.** NFATc1 correlated with IRF4 in PHA stimulated PBMCs in AG in the PreDicta study, n=7. **F.** NFATc1 mRNA was correlated with NFATc2 mRNA in PHA stimulated PBMCs of the AG (asthma group) of PreDicta n=8. Significance as indicated: *P ≤ 0.05; **P≤ 0.01; ***P ≤ 0.001

**Figure S2. A. NFATc1 and NFATc2 correlation with PD1 and Foxp3 in primary school children. A.** *NFATc1* and *NFATc2* and *IRF4* mRNA were measured in untreated PBMC culture in the cohort of asthmatic primary school children AGENDAs. **A.** NFATc1: AG: n=5; NFATc2: AG n=4. **B.** NFATc2 mRNA did not correlate with IRF4 mRNA in AG n=4. **C.** TGFβ1 expression measured by ELISA in unstimulated PBMCs in CG n=6 and AG n=4 **.** **D.** NFATc1 correlated with PD1 measured in untreated PBMC of the CG n=5 **E.** NFATc1 correlated with PD1 measured in untreated PBMC of the AG n=5 **F.** NFATc2 correlated with PD1 measured in untreated PBMC of the CG n=4 **G.** NFATc2 correlated with correlated with PD1 measured in untreated PBMC of the AG n=4. **H, I.** NFATc1 were correlated with FoxP3 measured in untreated PBMC of the CG n=7 and AG n=5.**J, K**.NFATc2 correlated with FoxP3 measured in untreated PBMC of the CG n=6 and AG n=4. Significance as indicated: *P ≤ 0.05; **P≤ 0.01; ***P ≤ 0.001

**Figure S3. Correlation of NFATc1 and NFATc2 with peripheral blood neutrophils, basophils and monocytes A.** NFATc1 was correlated with neutrophils in peripheral blood of the CG n=6 and **B.** AG n=4. **C, D.** NFATc2 correlated with neutrophils in peripheral blood of the CG n=7 and AG n=4. **E-H.** NFATc1 and NFATc2 were correlated with basophils in peripheral blood of the CG n=7 and AG n=4. **I-L.** NFATc1 and NFATc2 were correlated with monocytes in peripheral blood of the CG n=7 and AG n=4. Significance as indicated: *P ≤ 0.05; **P≤ 0.01; ***P ≤ 0.001
