# Supplementary material for: Role of nuclear factor of activated T cells 2 (NFATc2) in allergic asthma
Source: Immun Inflamm Dis. 2020 Oct 20;8(4):704–12. doi: 10.1002/iid3.360 (PMC7654396; doi:10.1002/iid3.360)
Supplement: Supplementary file 5 — Supporting information. [file IID3-8-704-s005.docx]

**Table S1. CAMP and Asthma BRIDGE cohorts’ Characteristics**

|  | Asthma (N=865) | No asthma (N=116) |
| --- | --- | --- |
| Study cohort  CAMP  ABRIDGE | 620  245 | 0  116 |
| Age (mean ± SD) | 12.5 ± 6.81 | 26.5 ± 1.73 |
| Gender  Female  Male | 359  506 | 70  46 |
| Race  White  African American  Other | 457  236  172 | 31  67  18 |

**Table S2: Clinical data on the allergy sensitization of the primary school-aged children of the AGENDAS cohort**

|  | Control Children | Asthma Children |
| --- | --- | --- |
| Number of subjects | 7 | 6 |
| Gender | 1(M)=14,35%  6(F)=85,7% | 3(M)=50%  3(F)=50% |
| Age | 8,36 | 7,73 |
| RAST | 6 RAST negative= 85,7%  1 RAST positive=14,14% | 3 RAST negative= 50%  3RAST positive= 50% |
| Daily treatment with IC* | 0 | 83,3%(5/6) |
| Dermatophagoides pteronyssinus | 14,3%(1/7) | 33,3%(1/6) |
| Birch | 0 | 50%(3/6) |
| Phleum | 0 | 33,3%(2/6) |
| Ambrosia | 0 | 16,7%(1/6) |
| Cat epithelium | 0 | 16,7%(1/6) |
| Dog epithelium | 0 | 16,7%(1/6) |
| FEV1/VC before bronchodialation** | 86,8 | 85,5 |
| FEV1/VC after bronchodialation** | 88,80 | 88,85 |
| Allergic rhinitis | 0 | 16,7%=(1/6) |
| Atopic eczema | 0 | 16,7%=(1/6) |
| Allergic rhinitis and atopic eczema | 0 | 0 |
| Without allergic commorbidity | 85,7%(6/7) | 50%(3/6) |
| Family predispositon to asthma | 0 | 66,7%(4/6) |
| Family predisposition to atopy | 57,1%(4/7) | 66,7(4/6) |
| Both parents with atopy | 0 | 50%(3/6) |
| Without family predispositions | 42,9%(3/7) | 33,3%(2/6) |

* inhaled corticosteroids

**FEV1 (%)= Forced expiratory volume in 1 second compared to vital capacity

B= Birch, L= Lieschgras/Pleum pratense, At= Alternaria tenuis, Hdm= House dust mites A= ambrosia, Pn= Penicillium notatum, Ch= Cladosporium herbarum, Af= Aspergillus fumigatus, Ce= Cat epithelium, De= Dog epithelium

Not measured because of lack of either blood (306) or material

Allergic senzitisation only at RK1 and above

Table S3: Clinical characteristics of the control group of the AGENDAS cohort at recruitment

| Control | FEV1  [%] | Seasonal asthma | Month of recruitment | RAST | NFATc1  /HPRT untr. | NFATc2  /HPRT untr. |
| --- | --- | --- | --- | --- | --- | --- |
| 1 | 94.1 | Neg | Apr | HDM RK6/>100 | 1,96 | 6,26 |
| 2 | 80 | Neg | Jan | neg | 1,34 | 3,33 |
| 3 | 71,7 | Neg | Jan | neg | 0,99 | 0,44 |
| 4 | 84,7 | Neg | Jan | neg | 1,13 | * |
| 5 | 94,1 | Neg | Jan | neg | 0,77 | 0.02 |
| 6 | 92,9 | Neg | Ma | neg | 0,52 | 2,02 |
| 7 | 90 | Neg | Jul | A RK0 / 0,03  B RK0 / 0,01  HDM RK0 / 0,03 | 0,86 | 2,53 |
| Mean | 86,8 |  |  |  | 1,08 | 2,43 |
| SEM | 8,5 |  |  |  | 0,18 | 0,92 |

**Table S4: Clinical characteristics of the asthma group of the AGENDAS cohort at recruitment**

| Asthma | FEV1[%] | Seasonal asthma | Month of recruitment | RAST kU/l | NFATc1  /HPRT untr. | NFATc2  /HPRT untr. |
| --- | --- | --- | --- | --- | --- | --- |
| 8 | 90,7 | Jan - Dec | Jan | B RK1/0.53  L RK1/0.48 | 2,93 | 3,40 |
| 9 | 85,5 | Nov -Feb | Feb | B RK1/0.46  AT RK2/0,87  HDM RK5/ 82,1 | 2,14 | * |
| 10 | 85 | Jan - Dec | Apr | neg | 2,51 | 8,36 |
| 11 | 89,4 | Oct - Apr, Jun | Jul | neg | 2,41 | 7,77 |
| 12 | 77,4 | Apr - Aug, Nov - Dec | Dec | A RK3 / 7,98  B RK3 / 9,91  L RK6 / >100  PN RK2 / 0,98  CH RK1 / 0,46  AF RK2 / 2,85  AT RK4 / 40,7  CE RK2 / 0,75  DE RK2 / 0,77 | * | * |
| 13 | 85,2 | Nov - Dec | Oct | A RK0 / 0,01  B RK0 / 0,01  L RK0 / 0,01  HDM RK0 / 0,01  CE RK0 / 0,01  DE RK0 / 0,01 | 1,16 | 9,07 |
| Mean | 85,5 |  |  |  | 2,23 | 7,15 |
| SEM | 1,9 |  |  |  | 0,30 | 1,27 |

**Table S5: White blood cell count of the AGENDAS Childrem**

|  | **Subject** | **Leucocytes** | **Neutrophils%** | **Eosinophils %** | **Basophils%** | **Lymphocytes%** | **Monocytes%** | **Neutrophils abs.** | **Eosinophils abs** | **Basophils**  **abs** | **Lymphocytes abs** | **Monocytesabs** | **IgE** |
| --- | --- | --- | --- | --- | --- | --- | --- | --- | --- | --- | --- | --- | --- |
| C | 303 | 9,25 | 41,3 | 12 | 0 | 37,7 | 8,5 | 3,8 | 1,12 | 0,04 | 3,5 | 0,8 | 402 |
|  | 307 | 9,85 | 58,1 | 2 | 0 | 33,1 | 6,4 | 5,72 | 0,18 | 0,04 | 3,26 | 0,63 | 35 |
|  | 308 | 6,53 | 60 | 3 | 0 | 29,9 | 6,7 | 3,92 | 0,18 | 0,02 | 1,95 | 0,44 | 266 |
|  | 309 | 6,8 | 43,4 | 3 | 1 | 47,6 | 5,4 | 2,94 | 0,18 | 0,05 | 3,24 | 0,37 | 16 |
|  | 310 | 7,88 |  | 0 | 1 | 28 | 5 |  | 0 | 0,08 | 2,21 | 0,39 | 3 |
|  | 311 | 8,92 | 72 | 2 | 0 | 18,4 | 7,4 | 6,42 | 0,14 | 0,03 | 0,64 | 0,66 | 43 |
|  | 312 | 5,93 | 38,4 | 4 | 1 | 50,9 | 6,2 | 2,27 | 0,22 | 0,05 | 3,02 | 0,37 | 59 |
|  | Mean | 7,88 | 52,20 | 3,71 | 0,43 | 35,09 | 6,51 | 4,18 | 0,29 | 0,04 | 2,55 | 0,52 | 117,71 |
|  | SEM | 2,98 | 21,31 | 1,40 | 0,16 | 13,26 | 2,46 | 1,71 | 0,11 | 0,02 | 0,96 | 0,20 | 44,49 |
| A | 301 | 7,31 | 37,1 | 1 | 1 | 51,2 | 10 | 2,7 | 0,09 | 0,04 | 3,7 | 0,7 | 96 |
|  | 302 | 8,38 | 54 | 6 |  | 30 | 9 |  | 0,5 |  | 2,5 | 0,8 | 1510 |
|  | 304 | 6,42 | 37,4 | 6 | 1 | 45,6 | 10,6 | 2,4 | 0,35 | 0,06 | 2,9 | 0,7 | 21 |
|  | 305 | 5,41 | 39,3 | 1 | 0 | 45,5 | 13,9 | 2,1 | 0,06 | 0,01 | 2,5 | 0,8 | 7 |
|  | 306 | 8,77 | 44,8 | 6 | 1 | 41,2 | 7,3 | 3,93 | 0,52 | 0,04 | 3,61 | 0,64 | 2003 |
|  | 313 | 7,8 | 47,9 | 6 | 0 | 39,7 | 6,4 | 3,73 | 0,45 | 0,01 | 1,1 | 0,5 | 577 |
|  | Mean | 7,35 | 43,42 | 4,33 | 0,6 | 42,2 | 9,53 | 2,97 | 0,33 | 0,03 | 2,72 | 0,69 | 702,33 |
|  | **SEM** | 3,00 | 17,72 | 1,77 | 0,27 | 17,23 | 3,89 | 1,33 | 0,13 | 0,01 | 1,11 | 0,28 | 286,73 |
